# Evolutionary cognitive therapy versus standard cognitive therapy for depression: a protocol for a blinded, randomized, superiority clinical trial

**DOI:** 10.1186/1745-6215-15-83

**Published:** 2014-03-19

**Authors:** Cezar Giosan, Oana Cobeanu, Cristina Mogoase, Vlad Muresan, Loretta S Malta, Katarzyna Wyka, Aurora Szentagotai

**Affiliations:** 1 Department of Clinical Psychology and Psychotherapy, Babes-Bolyai University, Cluj-Napoca, Romania; 2 Berkeley College, New York, NY, USA; 3 City University of New York, New York, NY, USA; 4 VISN 2 Upstate New York Health Care, Samuel S. Stratton VA Medical Center, PTSD/Combat Readjustment Program, Albany, NY, USA

**Keywords:** Depression, Protocol, Randomized trial, Evolutionary psychotherapy, Darwinian psychotherapy, Evolutionary psychology, Cognitive therapy, CBT, Cognitive evolutionary therapy

## Abstract

**Background:**

Depression is estimated to become the leading cause of disease burden globally by 2030. Despite existing efficacious treatments (both medical and psychotherapeutic), a large proportion of patients do not respond to therapy. Recent insights from evolutionary psychology suggest that, in addition to targeting the proximal causes of depression (for example, targeting dysfunctional beliefs by cognitive behavioral therapy), the distal or evolutionary causes (for example, inclusive fitness) should also be addressed. A randomized superiority trial is conducted to develop and test an evolutionary-driven cognitive therapy protocol for depression, and to compare its efficacy against standard cognitive therapy for depression.

**Methods/design:**

Romanian-speaking adults (18 years or older) with elevated Beck Depression Inventory (BDI) scores (>13), current diagnosis of major depressive disorder or major depressive episode (MDD or MDE), and MDD with comorbid dysthymia, as evaluated by the Structured Clinical Interview for DSM-IV (SCID), are included in the study. Participants are randomized to one of two conditions: 1) evolutionary-driven cognitive therapy (ED-CT) or 2) cognitive therapy (CT). Both groups undergo 12 psychotherapy sessions, and data are collected at baseline, mid-treatment, post-treatment, and the 3-month follow-up. Primary outcomes are depressive symptomatology and a categorical diagnosis of depression post-treatment.

**Discussion:**

This randomized trial compares the newly proposed ED-CT with a classic CT protocol for depression. To our knowledge, this is the first attempt to integrate insights from evolutionary theories of depression into the treatment of this condition in a controlled manner. This study can thus add substantially to the body of knowledge on validated treatments for depression.

**Trial registration:**

Current Controlled Trials ISRCTN64664414

The trial was registered in June 2013. The first participant was enrolled on October 3, 2012.

## Background

Depression affects around 350 million people worldwide, and is estimated to become the leading cause of disease burden globally by 2030 [1]. More than 30 million people are affected by depression in Europe alone, and the associated annual costs are estimated at €92 billion [2].

There are several existing effective treatments for depression, including cognitive behavioral therapy (CBT) [3-5], interpersonal therapy (IPT) [6-10] and antidepressants [11].

CBT is the psychological standard of care for depression recommended as the first-line psychotherapeutic intervention [12]. In CBT, the root of the depressive symptoms is seen as a set of dysfunctional core beliefs/schemas, which distort information processing, thus leading to systematic negative cognitive biases [3-5]. In CBT, the therapist tries to modify these dysfunctional beliefs, as well as engage the patient in rewarding activities.

IPT for depression focuses on ameliorating interpersonal difficulties and emphasizes the role of social support in the onset and maintenance of depression. The treatment rationale in IPT is that improvement in interpersonal relationships leads to improvement in depressive symptoms, which positively feeds back onto interpersonal functioning [13]. Like CBT, IPT is one of the recommended treatments of choice by the National Institute for Clinical Excellence [12] and the National Institute of Mental Health [14].

Research on the efficacy of the therapies for depression shows that 30% to 50% of the patients do not respond to anti-depressive medication [11], and around 40% of patients with moderate to severe depression do not respond well to CBT [15]. Moreover, 40% of those patients whose depression does remit will relapse within 2 years [16] and 20% are at risk of developing chronic, unremitting depression [16,17]. Generally, meta-analyses indicate that all *bona fide* (non-placebo) psychological treatments for depression are equally efficacious [18], and the effect sizes of psychotherapy for adult depression vary from small to moderate [19].

Therefore, despite existing efficacious treatments, efforts should be made to develop and provide improved evidence-based treatment protocols for patients with depression. These efforts should build on the already validated treatment approaches and strive to add incremental benefits by targeting additional factors potentially involved in the onset or maintenance of depression. The current clinical trial is designed to contribute to this desired outcome.

### Framework and rationale of the present study

Human behavior generally revolves around a finite set of biosocially relevant themes, and focuses on fitness maximization (that is, reproductive success). Biosocial goals are behaviors that increase fitness or that historically have increased fitness. Examples of these are satisfactory outcomes in areas such as shelter and security, nutrition and food acquisition, health, sexuality, mate selection, attraction, protection and retention, parenting, and in-group and between-group interactions [20]. Evolutionary psychologists have noted that when humans are successful at meeting these fitness-enhancing goals, they generally experience well-being and happiness [21]. On the other hand, failure (perceived or real) to meet these challenges results in dissatisfaction, depression, tension, or frustration [21].

A growing number of researchers are proposing that, in addition to targeting proximal or immediate causes of depression (for example, dysfunctional beliefs, as targeted by CBT), barriers to fitness should also be addressed. These factors are thought to be distal or evolutionary causes of depression.

In one of the early attempts at evolutionary explanations for depression, Dobson and Show [22] argued that Beck’s cognitive distortions are a consequence of depression, rather than a cause of it. Recent research has provided more empirical support for these insights, which link threats to fitness to depression. For example, a large study conducted on a sample of 1,400 disaster workers found that a successful slow fitness strategy (High-K) was a negative predictor of a variety of psychopathologies, including post-traumatic stress disorder, functional disability, anger, and sleep disturbances [23]. Another study showed that the same fitness strategy was an important negative predictor of depressive symptomatology, accounting for 15% of the variance in scores on the Beck Depression Inventory, Second Edition (BDI-II), [24] after controlling for risk factors for depression [25]. Elsewhere, greater depressive symptomatology scores have been shown to correlate negatively with ratings of mate value for self, and indirectly predicted lower ratings of mate value for short-term and long-term partners, as well as best friends [26].

These findings support a relationship between fitness level and depressive symptomatology, and suggest that interventions that specifically target increasing fitness could potentially alleviate depression.

### Objectives

It is the goal of the present study to explore the efficacy of formally incorporating interventions on the distal causes of depression in the treatment of this condition. An evolutionary-driven cognitive therapy (ED-CT) protocol for depression is being developed and tested in this clinical trial. Thus, patients with depression initially complete a questionnaire aiming at identifying deficient areas of fitness, and are guided by the therapist on ways of improving on those areas. The therapeutic outcome will be compared with the outcome of a group of patients with depression undergoing a well-established CBT treatment, namely, Beck’s cognitive therapy (CT) for depression [27].

### Trial design

This study is designed to be a blinded, randomized, superiority clinical trial. The experimental group receives ED-CT, while the control group receives CT. We hypothesize that, because the intervention array is larger in the case of ED-CT, the therapeutic gains will be larger in this condition than in the CT group at the completion of the therapy, and that this will be maintained at the 3-month follow-up.

## Methods

### Study setting

The study population consists of Romanian adults with a diagnosis of major depression as defined in the *Diagnostic and Statistical Manual of Mental Disorders*, Fourth Edition, Revised Text (DSM-IV-TR) [28], or with depressive symptomatology. The study is being implemented through the Babes-Bolyai University Psychological Clinic, Cluj-Napoca, Romania.

#### Eligibility criteria

Romanian-speaking adults (18 years or older) with raised BDI [24,29]) scores (>13), current diagnosis of major depressive disorder or major depressive episode (MDD or MDE), and MDD with comorbid dysthymia, are included in the study. Participants presenting with panic disorder, substance abuse, psychotic symptoms, organic brain disorders (for example, dementia), imminent risk of suicide, self-injury or harming others, or serious legal or health issues that would prevent from regularly attending, are excluded.

### Interventions

#### The CT group

The CT group undergoes a 1-hour session of CT [27] each week for 12 weeks. CT is a structured, goals-oriented, short-term intervention organized around the concepts of automatic thoughts and schema/core beliefs. A distinctive feature of CT is the cognitive conceptualization of the client’s problems. Problematic behaviors are seen as consequences of the dysfunctional thinking patterns. Therefore, clients are trained to identify and modify dysfunctional automatic thoughts and beliefs. When behavioral techniques are used, they are employed in the service of cognitive change (for example, behavioral experiments to challenge dysfunctional thoughts), as well as to increase the number of positive reinforcements. The treatment begins with the identification of negative automatic thoughts (conceptualized as the most easily accessible cognitive contents, as they are linked to specific activating events), followed by the examination of their validity and utility, to construct a more adaptive viewpoint and thus decrease depressive symptoms. As the therapy progresses, deeper cognitive structures are targeted (including underlying assumptions/intermediate beliefs and dysfunctional schemas), with the ultimate goal of helping the client to achieve a remission of their depression and prevent relapse.

#### The ED-CT group

The ED-CT group also undergoes a 1-hour session of therapy each week for 12 weeks. Therapy sessions follow the guidelines of the ED-CT manual that we have developed. Following implementation of the study, the manual will be available upon request from the first author, and will also be submitted for publication.

The ED-CT starts with the administration of a Fitness Evaluation Scale (FES), described below, which yields a comprehensive assessment of the client’s perceived darwinian or evolutionary fitness. The therapist thus begins the treatment with a clear understanding of the barriers to fitness that the patient is facing, in addition to the problems formulated by them at the beginning of therapy. In other words, the patient’s answers to the FES guide the evolutionary-driven intervention elements by providing additional focuses for treatment, alongside classic CT targets of intervention. Thus, the key difference between ED-CT and a classic CT approach is that in the ED therapy, the therapist comes pre-equipped with a list of fitness deficiencies identified by the patient through the completion of the FES at intake, which the therapist addresses alongside other problems.

The ED-CT intervention is based on standard CT while adding specific goals and techniques designed to increase an individual’s evolutionary fitness. We will succinctly describe the treatment course, focusing on the evolutionary aspects of the treatment.

The first treatment session focuses on educating the patient about typical topics such as depression and psychotherapy, emphasizing the importance of ‘homework’ (personal practice of the therapy at home), taking responsibility for change, and adjusting the patient’s expectations. This session also focuses on presenting details about specific ED-CT insights such as the mismatch theory (the theory that cognitive structures which were adaptive in a Pleistocene environment, but are now ‘mismatched’ to the current environment, lead to dysfunctional emotions and behaviors), and about the hypothesized evolutionary functions of depression [30]. The patient is informed in this session about the research linking depression and fitness-related behaviors, and why an evolutionary approach can be suited as a guiding add-on to a CT intervention. This is also the point at which the therapist discusses the problems identified by the FES at intake, and, with the help of the patient, sets realistic goals to address them in future sessions. The homework in this session usually consists of building specific goals, using the FES as a guideline and as an example of specific target behaviors.

Session 2 focuses on continuing to build the therapeutic alliance and elaborating on the fitness problems list yielded by the FES. This session also provides the opportunity to discuss with the patient their understanding of the distal evolutionary causes of depression.

Sessions 3 to 6 focus on working on each of the fitness deficiencies identified at intake. These problems are conceptualized using evolutionary insights. For instance, some dysfunctional beliefs and their consequences can be seen as adaptive mental structures left over from the Environment of Evolutionary Adaptedness (EEA) [31]. This helps the patient to experience control and self-acceptance of their depressive symptoms. For example, a patient can present guilt – a depression symptom – about feelings or expressions of anger, fueled by beliefs such as: ‘I’m a horrible person because I got angry and yelled at my neighbor.’ In this case, guilt is typically seen as a secondary emotional problem (dysfunctional emotion about other emotions) [32]. Evolutionary explanations of anger (for example, anger as an adaptive reaction during the EEA) can lower self-criticism and promote self-acceptance by reducing the intensity of the secondary emotion (guilt in this example), thus enabling the psychotherapist to work on the primary, present problem (anger in this example).

Homework is negotiated during these sessions, and generally consists of self-monitoring of emotions and behaviors, cognitive restructuring, and behavioral activation tasks generated from the FES. Specific behaviors that the patient does not engage in as captured by the FES (for example, spending time in nature, healthy eating habits) are targeted for activation. In addition, problem-solving, conflict resolution, and assertiveness training are used to enhance relationships with the patient’s relatives where appropriate, thus tapping into indirect fitness, while direct fitness (for example, mate value, health, income) is enhanced with standard CT techniques.

Sessions 7 to 10 are targeted at making headway towards the resolution of some or all of the problems previously identified by the FES, working toward strengthening the patient’s adaptive beliefs and weakening the maladaptive beliefs, and encouraging them to see the links between problems, particularly those that are characterized by common dysfunctional beliefs. Core beliefs are identified and approached in this part of the therapy. Standard CT techniques are used to change beliefs and behaviors, but the therapist often refers back to the evolutionary-informed CT conceptualization. Homework continues to focus on FES-generated behavioral activation, rehearsing adaptive statements in real-life situations, and applying the techniques and conceptualization to novel, diverse problems.

Sessions 11 and 12 prepare the patient for the task of becoming their own future therapists, and for discussing relapse prevention. The homework focuses on strengthening confidence in healthy core beliefs and promoting self-acceptance and other-acceptance. Self-control techniques in difficult situations are practiced, and solutions for possible relapses are tested before the end of the therapy.

### Outcomes and measures

#### Primary outcomes

The level of depressive symptomatology and a categorical diagnosis of depression after treatment constitute the primary outcomes.

#### Secondary outcomes

Perceptions about quality of life and social functioning constitute the secondary outcomes.

Because the interventions in the experimental group target both proximal and distal causes of depression, it is expected that they will lead to a greater decrease in depressive symptomatology and a greater increase in quality of life and in social and occupational functioning than those in the control group.

#### Other outcomes

We considered three sets of additional outcomes, as follows.

The first set comprises measures related to fitness and mate value; self-reported physical health; behavioral activation; self-reported religiosity; self-reported coping strategies; and measures of positive and negative emotions. We decided to include these measures to investigate, in an exploratory manner, if the eventual superiority of the ED-CT intervention is related to improvements in fitness dimensions (for example, kinship relations, upward mobility, physical health, social capital) We also included behavioral activation as a potential predictor of outcomes for both interventions [27,33]. In addition, we were interested to see if the ED-CT intervention efficacy is associated with a person’s religiosity, as a high level of religious faith might undermine the acceptance of the ED-CT intervention rationale. Lastly, we included measures of emotionality and coping strategies to explore the extent to which the newly devised intervention might have broader clinical implications.

The second set of additional outcomes includes measures of presumed mechanisms of change within classic CBT (including CT): negative automatic thoughts [3], dysfunctional attitudes [3,34], and irrational beliefs [35,36]. We included these measures to examine, in an exploratory manner, if the two interventions have differential effects on the same cognitive mechanisms.

The third set of additional outcomes includes measures of working alliance, treatment outcome expectancies, and client satisfaction with the therapy. We added these measures to allow exploratory investigation of the eventual differences between the two interventions in terms of non-specific factors associated with the treatment outcome.

The instruments that we use for measuring each of these outcomes are briefly described below.

#### Measures of primary outcomes

The Structured Clinical Interview for DSM-IV (SCID) [37], specifically, the Overview, Mood Episodes, and Anxiety Disorders modules, is used for the clinical assessment of depression. The Overview module collects information about sociodemographic variables (date of birth, marital status, number of children, level of education, employment status), drug use, physical and psychological treatment history (including any past or current treatments for depression), and current social functioning. The Mood Episodes and Anxiety Disorders modules follow the diagnostic criteria of the DSM-IV-TR [28] for mood episodes and anxiety disorders. The SCID has been adapted for use on the Romanian population [38].

The BDI-II [24,29], a 21-item version of the original BDI [39], is one of the most widely used self-report measures of depression. Participants rate the intensity of depressive symptoms on a Likert scale from 0 to 3, where 0 corresponds to lack of the symptom, while 3 corresponds to the highest intensity. The psychometric properties of the original BDI are well established, and the BDI-II also appears to be psychometrically strong [24]. The BDI-II has been adapted for use with the Romanian population [40], and has adequate psychometric properties [41].

### Measures of secondary outcomes

The Social Adjustment Scale (SAS) [42-44] is a self-report measure of social functioning used in both research and clinical practice. The questions are designed to assess six key role areas: 1) work, 2) social and leisure activities, 3) relationships with extended family, 4) role as a marital partner, 5) parental role, and 6) role within the family unit. The items are coded on a five-point Likert scale. An overall score is obtained, as well as scores for each of the six role areas [45]. Larger scores denote greater impairment. The SAS has been translated into Romanian and adapted for the purposes of this study, as described further below.

The World Health Organization Quality of Life BREF (WHOQOL-BREF; [46]) is a 26-item self-report tapping into the following broad domains: physical health, psychological health, social relationships, and environment. The Cronbach’s α values for these domains are acceptable, varying from .68 to .81 [47-49]. Items are rated on a five-point Likert scale and scored in a positive direction (that is, higher scores indicate higher quality of life). The scale has been translated and adapted into Romanian for the purposes of the current study.

### Measures of other outcomes

For the first set of additional outcomes used in this study, the following measures are used.

• The FES is a scale adapted and expanded by the authors from the High-K Strategy Scale (HKSS) [50]. The HKSS has been shown to be negatively associated with depressive symptomatology [25] and psychopathology in general [23]. The FES consists of 45 items (58 if the patient has children), tapping into various dimensions theorized to make up the indicators of fitness. Participants rate every item on a five-point Likert scale. A total FES score is computed by adding up ratings for each item, with higher scores indicating greater fitness. The FES was preliminarily validated on a sample of 146 subjects, and showed good internal consistency (Cronbach’s α = 0.93). The FES is the therapist’s starting point in prescribing the evolutionary-driven interventions, as further detailed below.

• The Mate Value Inventory (MVI) [26] is a multivariate assessment of attributes desired in the self, and in social or sexual partners.The MVI is a list of 17 traits, for which participants are asked, ‘How well do you feel that these attributes apply to you currently,’ and are measured on a scale of -3 (extremely low on this trait) to +3 (extremely high on this trait). These traits are: ambitious, attractive face, attractive body, desires children, emotionally stable, enthusiastic about sex, faithful to partner, financially secure, generous, good sense of humor, healthy, independent, intelligent, kind and understanding, loyal, responsible, and sociable. Mate value is the summed score of these items. The MVI has been translated and adapted into Romanian for the purposes of the current study.

• The Components of Mate Value Survey (CMVS) [51] is a 21-item self-report measure. It is based on the Self-Perceived Mating Success Scale (SPMSS) [52], but in addition to the aspects of mate value indexed by SPMSS, it includes aspects related to sociality, parenting, wealth, looks, relationships history, and fear of failure. The CMVS has adequate reliability [26], and has been adapted into Romanian for the purposes of this study.

• Behavioral Inhibition System/Behavioral Activation System (BIS/BAS) [53] is a 20-item self-report questionnaire that consists of a Behavioral Inhibition subscale (making up the BIS) and three Behavioral Activation subscales (making up the BAS). Each item is rated on a four-point Likert scale, from 1 (strongly agree) to 4 (strongly disagree). A number of studies have indicated adequate psychometric properties [54,55]. The BIS/BAS scales have been validated on the Romanian population in previous studies [55].

• The Physical Health Questionnaire (PHQ) [56] is a brief 14-item self-report scale used to assess frequency of somatic symptoms (gastrointestinal problems, headaches, sleep disturbances, and respiratory problems). Items 1–11 are rated on a seven-point frequency scale, ranging from 1 (not at all) to 7 (all of the time). Items 12–14 have different frequency-related responses, but the participant uses the same seven-point frequency scale. Higher mean scores reflect better somatic health. The PHQ has shown good psychometric properties [57-60], and has been translated into Romanian for the purposes of this study.

• The Santa Clara Strength of Religious Faith Questionnaire (SCSRFQ) is a brief, reliable and valid self-report measure assessing strength of religious faith and engagement, which is suitable for use with multiple religious traditions. It comprises 10 items, scored on a four-point Likert scale, with higher scores denoting increased levels of religious faith. The SCSRFQ has been widely used in medical, student, psychiatric, and general populations internationally, and has good psychometric properties [61-65]. The questionnaire has been translated and adapted into Romanian for the purposes of this study.

• Brief COPE (B-COPE) [66] is a 28-item brief form of a previously published measure called the COPE inventory [67], which has proven to be useful in health-related research. Respondents rate the items on a four-point Likert scale, from 1 (I haven’t been doing this at all) to 4 (I’ve been doing this a lot). Scores are derived separately for two main categories of coping strategies, namely, ‘emotion focused’ and ‘problem focused’ coping. B-COPE has been successfully used on Romanian samples [40].

• The Positive and Negative Affect Scale (PANAS) [68] is a 20-item self-report questionnaire, designed to assess mood. It distinguishes between positive and negative affect. Participants rate each item on a five-point Likert scale, from 1 (very slightly/not at all) to 5 (extremely) to indicate how they felt during the indicated timeframe. The PANAS can be used to assess mood on various time scales by altering the instructions. Possible time scales include moment, today, past few days, past week, past few weeks, past year, and general. For the purposes of this study, we used a 2-week interval. The validity and internal consistency of the PANAS are good, with test–retest reliability being the highest for the ‘general’ temporal instruction [68]. The PANAS has been used previously on the Romanian population, and was found to have adequate psychometric properties [69,70].

To explore the different mechanisms by which the two treatment approaches (ED-CT and CT) might lead to improvement (that is, the second set of additional outcomes), the following measures are administered.

• The Automatic Thoughts Questionnaire (ATQ) [71] is a 15-item self-report measure used to assess depression-related cognitions. Participants rate on a five-point scale, (from 1 (never) to 5 (almost all the time)) how frequently they have had a given thought over the past week. A higher score shows a higher frequency of automatic thoughts. The psychometric properties of the ATQ have been adequately demonstrated in previous studies [72].

• The Dysfunctional Attitudes Scale (DAS) [73] is a 40-item self-report instrument which measures attitudes that, according to the cognitive theory of depression, contribute to vulnerability for depression. Participants rate on a seven-point scale, from 7 (total disagreement) to 1 (total agreement), the level of personal concordance with the item presented. The higher the score, the more dysfunctional the participant’s attitudes. Adequate internal consistency and test-retest reliability for DAS have been reported in the literature [74].

• The General Attitudes and Beliefs Scale (GABS) [75] is a 26-item self-report instrument that measures irrational beliefs related to six different content areas: achievement, approval, comfort, justice, self, and others. In addition, the scale includes a short index of rational beliefs. Participants rate on a five-point scale the level of agreement or disagreement with the item presented, from 1 (strongly disagree) to 5 (strongly agree). Adequate psychometric properties have been reported in the literature [75,76].

• The Attitude and Beliefs Scale II (ABS II) [76] is a 72-item instrument that measures irrational cognitions (that is, demandingness, global evaluation/self-downing, low frustration tolerance, and awfulizing) shown to be involved in the onset and maintenance of emotional distress, as well as their rational counterparts (preferential thinking, unconditional self-acceptance, frustration tolerance, and non-awfulizing). Each item is rated on a five-point Likert scale, from 0 (strongly disagree) to 4 (strongly agree). A total irrationality score is obtained by summing up the scores on each irrationally worded item, and the reverse scored responses on each rationally worded item. Higher scores indicate a higher level of irrationality.

All the instruments in the second set of additional outcomes have been successfully used previously with the Romanian population (ATQ [77-79]; DAS [78-80]; GABS [77-79]; ABS II [81-83]).

The third set of additional outcomes contains the following measures.

• The Working Alliance Inventor*y* (WAI) [84,85] is a 12-item self-report global measure of the therapeutic alliance. The WAI has three subscales: 1) emotional bond of trust and rapport between the counselor and client, 2) agreement about the overall goals of treatment, and 3) agreement about the tasks relevant for achieving these goals. Items are rated on a seven-point Likert scale and the scale has shown good internal consistency, as well as good construct, concurrent, and predictive validity [85-87]. The questionnaire has been translated and adapted into Romanian for the purposes of this study.

• Treatment outcome expectancies are measured using 10-cm visual analogue scales (VASs). This approach has shown good reliability in multiple studies [88,89]. Treatment outcome expectancies are measured at intake and at mid-treatment.

• The intent to attend the next session is also assessed using the Intent-to-Attend Scale [90], a single-item measure. Participants rate their intention on a nine-point Likert scale.

• After each therapy session, participants’ satisfaction with the treatment is evaluated using the Client Satisfaction Questionnaire [91-93], the eight-item version. The CSQ-8 assesses global patient satisfaction with a four-point Likert, scale and also provides a general score varying from 8 to 32. This questionnaire has been translated and adapted into Romanian for the purposes of this study.

All the instruments adapted into Romanian for the purposes of the present study were independently translated by two Romanian English-proficient PhD-level clinical psychologists with good knowledge of the constructs measured. Disagreements were resolved through discussions between the translators, and two Romanian senior clinical psychologists also reviewed and approved the final versions.

#### Participant timeline

For the study, potential participants are provided with verbal and written information during the first appointment with a clinical psychologist. Written consent for the participation in the clinical evaluation part of the study is sought at this time. A second appointment is scheduled, in which the respondents are evaluated clinically. If the inclusion criteria are met, written consent for participation in the treatment part of the study is obtained, within a 2-week interval.

**Figure 1:**
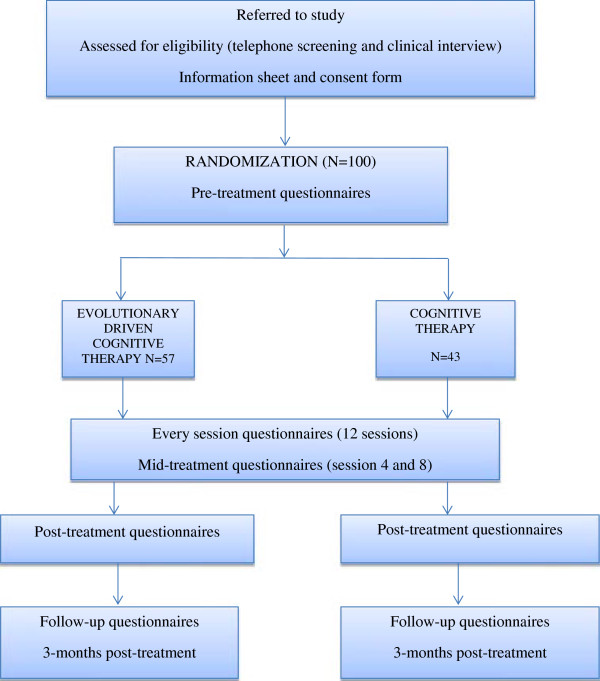
**CONSORT flow diagram**[94]**showing subject allocation to the therapy conditions.**

The participants are then randomized by a research assistant to receive either: 1) ED-CT or 2) CT, without being informed about their group allocation (see flowchart in Figure 1). Randomization is performed *a priori* using a computer software package (http://www.randomizer.org). In addition, the clinical psychologists who perform the clinical assessment are blinded to the experimental condition through the entire duration of the study. Participants who do not meet the criteria for the study are referred to the Babes-Bolyai Psychological Clinic or to a psychiatric clinic in Cluj-Napoca.

### Sample size

An *a priori* power analysis based on a medium effect size estimation indicated that a total of 100 participants are needed (planned main statistical test is ANOVA (repeated measures, between factors); *f* effect size: 0.247; statistical power: 0.80; α error probability: 0.05; two groups, two main measurements (pre-intervention and post-intervention); correlation between repeated measures: .50). Power analysis was computed using the G*Power 3.1.6 program [95]. Consequently, we randomized a total of 100 participants. Following generation of the random numbers, we decided to use the first sequence of numbers for the participants’ allocation to the groups. This resulted in 43 participants randomized to the CT group, and 57 participants randomized to the ED-CT group.

### Recruitment

An initial telephone interview screens out those people whose participation in the study is motivated by problems other than their mood. The potential subjects then undergo a clinical evaluation, performed by doctoral-level clinical psychologists certified by the Romanian Board of Psychologists, and currently supervised in their activity by senior psychologists. They have been trained in using the SCID [37] as part of their clinical program, at Babes-Bolyai University. Additionally, for the purposes of this study, all of them received specific practical training in administering the SCID modules used in the current protocol. To ensure the adequacy of the assessment procedure, all the initial clinical evaluations are audio recorded and randomly verified by a senior clinical psychologist. Moreover, all the potential eligible cases are discussed with the same senior clinical psychologist before enrolment. Every evaluation is audio recorded, to allow for reliability checks for depression diagnoses, performed by an independent evaluator, on 25% of randomly selected interviews. SCID [37] and BDI-II [24,29] (see above) are used for purposes of evaluating eligibility.

Prospective participants are recruited into the treatment study by clinical psychologists at the Babes-Bolyai University Psychological Clinic. Potential participants are also referred by psychiatric clinics that collaborate with Babes-Bolyai University as well as by private practices. Additionally, media avenues (for example, local newspapers, radio, social networks) as well as posters and fliers are used for recruitment purposes.

#### Assignment to interventions

The participants are randomly assigned to one of the two conditions using a sequence generated by the software Randomizer.org. Randomization is performed by a research assistant using a simple (unrestricted) randomization sequence that assigns two unique numbers per participant; the number assigned is either 1 or 2, according to the number of experimental conditions. To conceal the allocation mechanism, the same research assistant puts the patient in touch with their therapist without revealing anything about the intervention protocol to be implemented by that therapist.

The CT and ED-CT therapy protocols are each delivered by two different psychotherapists. All four psychotherapists are certified by the Romanian Board of Psychologists, and have extensive training in CT, completed through the Romanian Association of Cognitive and Behavioral Psychotherapies. The two psychotherapists delivering the ED-CT protocol have received additional training in the evolutionary components of the ED-CT protocol. Under the direct supervision of the principal investigator, the therapists have undergone 6 weeks of training, following the protocol manual for the ED-CT group.

The principal investigator and the statisticians running the data analysis will remain blinded to the experimental condition until the completion of the study.

### Data collection, management, and analysis

Each eligible patient is assigned a unique identification number and asked to complete the evaluation package, including the primary, secondary, and other outcomes, as well as demographic information (initial assessment). The same set of measures is administered after the final session (post-intervention assessment) and at 3 months after the final session (follow-up assessment). After sessions 4 and 8, patients also complete several measures of primary outcomes (that is, BDI-II), and other outcomes (ATQ, DAS, ABS II, PANAS, B-COPE, treatment outcome expectancies,WAI, CSQ-8, and Intent-to-Attend Scale). In addition, the participants complete the BDI-II, CSQ-8, and Intent-to-Attend Scale after each session.

To ensure high accuracy of data collection, all the measures are completed electronically, via a special system designed to reliably capture and organize the data points while minimizing the risk of missing responses. The system has been especially developed for the purposes of this study, and is secured on a server. Each participant uses their unique identification number to log on to the system. The therapists are responsible for assisting the patients in this process, and providing clarifications when needed.

To promote participant retention and follow-up completion, several strategies are being used. First, the participants receive a clear and complete description about the project, including, but not limited to, information regarding the type of psychotherapy offered, that is, CT, which is the standard of care in the psychological treatment for depression, having remission rates comparable with medication, and being more efficient than medication alone in the long term. The participants are also informed that the treatment is being provided free by therapists trained in the top-ranked Department of Clinical Psychology and Psychotherapy in the country [96], recently added, through its academic spin-off, the International Institute for the Advanced Studies of Psychotherapy and Applied Mental Health, to the Mapping of the European Research Infrastructure Landscape (MERIL) network [97]. Because in Romania psychotherapy sessions are not reimbursed by health insurance companies, we expect that this financial factor will also act as a motivator for the patient to stay in treatment. Furthermore, the therapy sessions are scheduled in a flexible manner, according to the patient’s preferences. Additionally, the therapist will call the participant the day before the planned session for a brief check, and to ensure continuation in therapy. Most importantly, to ensure retention, at the end of each session the participants complete the ‘Intend-to-Attend’ one-item scale, which is meant to promote commitment to the therapeutic process as well as to detect any issues that might require the therapist’s special attention. Lastly, the therapists carefully prepare the end session of the therapy and explain to the participants that they will keep in touch. The therapists also remind the patients at this time that the free treatment was possible because of a research grant, which makes the collection of follow-up measurements essential.

The severity of, and improvement in the BDI-II scores within and between the groups will be examined using mixed-effects linear regression analysis with a random intercept and slope over time (four assessments: baseline, after sessions 4 and 8, post-treatment) and fixed effects for treatment assignment.) The 3-month follow-up data will be analyzed in a separate mixed-effects model. The main analysis will compare groups in terms of depression level (that is, diagnostic status and symptom intensity). Results on the other measures will be investigated in an exploratory manner. When analysis of secondary/other outcomes are performed, the error probability will be adjusted according to the number of group comparisons performed [98].

### Monitoring study implementation

The management of unintended effects or harm (that is, clinically significant increase in depressive symptomatology, as measured by the BDI-II), is performed by the supervising clinical psychologist employed for this study, who monitors the clinical evaluations and therapy sessions. If necessary, the supervisor can decide to terminate the treatment/clinical assessment and make a further referral.

### Ethics and dissemination

The study has been approved by the ethics commission at Babes-Bolyai University. As with any study on depression, there are ethical concerns that need to be addressed. First, the exclusionary criteria exclude people who pose an imminent danger to themselves or others. These people will be immediately referred to the appropriate services dealing with such cases. Second, if a patient’s condition worsens during the therapy, the supervising clinical psychologist can opt to terminate the treatment ahead of schedule and make an appropriate referral. Third, people with mental conditions other than depression are also excluded, and will be referred to the services best suited to dealing with their problems.

One arm of this clinical trial involves interventions that stem from an evolutionary understanding of human behavior. The role of the SCSRFQ scale, administered at intake, is to assess the strength of religious beliefs before the first session, when the ED-CT conceptualization takes place. Special care will be taken with the patients whose religious beliefs might make them less receptive to the theoretical framework of such interventions. In such cases, the therapist will be mindful of the patient’s beliefs, and explain the basic tenets of ED-CT in a very sensitive manner.

Finally, confidentiality and privacy are of paramount importance. The completed forms of the clinical instruments are kept in locked cabinets and the access to the electronic data is password-protected. The passwords are changed regularly, and the clinical assessment reports contain no identifying information.

### Dissemination policy

The principal investigator and the research team intend to publish the trial results in a peer-reviewed journal, focusing on two main areas: 1) primary outcomes results and 2) mechanisms of change results. Presentations at international conferences on the topic are also envisioned. Furthermore, the intervention manual will be sent out for publication, and will be made available upon request from the first author.

## Discussion

This protocol describes a randomized trial comparing two interventions for depression: classic CT and the newly proposed ED-CT. As the costs of depression continue to mount [99], it is imperative that new approaches be tested and validated, and if successful, implemented. Given the high prevalence of depression and its projected costs, even incremental benefits of novel approaches can translate into billions of dollars in savings.

To our knowledge, this is the first attempt to integrate insights from evolutionary theories of depression into the treatment of this condition in a controlled manner. This study can thus add substantially to the body of knowledge on validated treatments for depression. The randomized controlled design is rigorous, allowing the authors to arrive at valid conclusions about the comparative efficiency of these two interventions at the end of the study.

There are, nonetheless, several caveats that must be acknowledged. As with any clinical trial testing psychotherapeutic interventions, therapist blinding is not possible. To minimize possible biases, the principal investigator will not be involved in any therapeutic sessions. In addition, because of the ethical concerns of keeping patients with depression on a waiting list for 12 weeks, the present study lacks a control group in that sense.

This randomized trial is a first step in the development of an evolutionary-driven intervention for depression. If the treatment is shown to be at least comparable with CT, then a future trial will examine whether this intervention is associated with lower relapse or other longer-term outcomes such as improved health.

### Trial status

Participant recruitment began on September 1, 2012. Randomization of the participants was performed on September 27, 2012. The first participant was enrolled on October 3, 2012.

## Abbreviations

ABS II: Attitude and Belief Scale II; ATQ: Automatic Thoughts Questionnaire; B-COPE: Brief COPE; BDI-II: Beck Depression Inventory, Second Edition; BIS/BAS: Behavioral Inhibition System/Behavioral Activation System; CMVS: Components of Mate Value Survey; CSQ-8: Client Satisfaction Questionnaire, 8-item version; CT: Cognitive Therapy; DAS: Dysfunctional Attitudes Scale; DSM-IV-TR: *Diagnostic and Statistical Manual of Mental Disorders*, Fourth Edition; ED-CT: Evolutionary-Driven Cognitive Therapy; FES: Fitness Evaluation Scale; GABS: General Attitudes and Beliefs Scale; MDD: Major Depressive Disorder; MDE: Major Depressive Episode; MVI: Mate Value Inventory; PANAS: Positive and Negative Affect Scale; PHQ: Physical Health Questionnaire; SAS: Social Adjustment Scale; SCSRFQ: Santa Clara Strength of Religious Faith Questionnaire; SCID: The Structured Clinical Interview for DSM-IV; WAI: Working Alliance Inventory; WHO: World Health Organization; WHOQOL-BREF: WHO Quality of Life BREF.

## Competing interests

The authors declare that they have no competing interests.

## Authors’ contributions

CG was responsible for study conception and design and manuscript writing; OC was responsible for manuscript writing and critical revision of the work; CM was responsible for manuscript writing and statistical analyses; VM was responsible for study conception, and critical revision of the work; LM was responsible for conception and design of the study, and critical revision of the work; KW was responsible for statistical analyses and critical revision of the work AS was responsible for study design and critical revision of the work. All authors read and approved the final manuscript.
